# Using Positive Empathy Interventions to Reduce Stigma Toward People Who Inject Drugs

**DOI:** 10.3389/fpsyg.2021.616729

**Published:** 2021-07-09

**Authors:** Alex J. Clinton, Robin A. Pollini

**Affiliations:** School of Medicine, West Virginia University, Morgantown, WV, United States

**Keywords:** stigma, empathy, intervention, drug-use, emotional exhaustion

## Abstract

People who inject drugs are often the target of stigma that puts this already at-risk group at greater risk of harm. Past research has shown that holding stigmatizing views of people who inject drugs increases risky behaviors and is a barrier to their engagement in important medical and public health interventions. One explanation is that the negativity surrounding the group causes increased levels of anticipated emotional exhaustion, discouraging positive engagement. However, there has been minimal research focused on addressing this negativity to reduce levels of held stigma against people who inject drugs. We hypothesized that giving people an imagined positive contact exercise about people who inject would lead to a reduction in stigma, since exposure to positive empathy may create new mental associations between stigmatized groups and more positive emotions and experiences. Secondarily, we hypothesized that positive empathy strategies would be more effective than traditional informational or learning based techniques, and that the latter would be more effective than a control condition. Our sample consisted of 375 participants recruited online. Participants were assigned to one of three study conditions: a positive empathy condition, an informational learning condition, or a control condition, and completed a posttest social distance measure. Results demonstrated that subjects exposed to the positive empathy stigma reduction condition experienced a significant reduction in held stigma while participants exposed to traditional informational learning techniques showed no significant reduction in held stigma. Positive empathy-based stigma interventions should be further researched as a promising avenue to reduce the effects of drug-related stigma.

## Introduction

Opioid use has increased substantially in the United States over the past decade, initially fueled by misuse of prescription opioids and more recently sustained by the use of heroin and fentanyl. Concurrent with this increase in opioid use, the United States has seen alarming increases in drug-related mortality and morbidities related to injection drug use including overdose fatalities, viral hepatitis, HIV, and serious injection-related bacterial infections like endocarditis (Zibbell et al., 2015; Hedegaard et al., 2020; Wong et al., 2020). There are effective medical treatments for opioid use disorder, as well as effective public health interventions to reduce overdose mortality and injection-related infections. However, there is growing evidence that stigma against people who inject drugs can serve as a barrier to access and utilization of these services. This stigma, defined in the current study as a negative association with a person as a result of a particular trait (Link and Phelan, 2001), has harmful effects on the group associated with these negative feelings and in many cases can predict negative health outcomes for that group, including difficulty seeking treatment for their substance use disorder.

Research by Paquette et al. (2018) found that after comprehensive interviews with a sample of people who inject drugs, many reported that stigma served as a substantial barrier to both substance use disorder treatment and medical services for injection-related infections. Similarly, earlier research demonstrates that stigma-related barriers to health services for people who inject drugs are associated with riskier drug use behavior (Latkin et al., 2010) and decreased likelihood to seek treatment for injection drug use (Keyes et al., 2010; Browne et al., 2016). In measuring the levels of held stigma, research has found that a representative sample of Americans desire social distance from people who inject drugs and that their drug use behavior scares them (Barry et al., 2014; Kolodny et al., 2015; Lang and Rosenberg, 2017). This stigma is even perpetuated among people who inject drugs, as research has shown that they may hold stigmatizing beliefs about themselves (Latkin et al., 2013; Silveira et al., 2018).

The literature also suggests that stigma against people who inject drugs is associated with negative health outcomes for this stigmatized population (Keyes et al., 2010; Latkin et al., 2010; Browne et al., 2016; Paquette et al., 2018) and their family members (Corrigan et al., 2006; McCann and Lubman, 2017). Ahern et al. (2007) examined the relationship between stigma against illicit drug users on mental and physical health. They found that discrimination and alienation predicted poorer mental health and that only discrimination was also associated with poorer physical health. Latkin et al. (2013) further examined the harmful effects of perceived drug user stigma and its relationship to depression among participants from Baltimore, MD with a history of recent drug use. The study showed that drug use stigma was significantly associated with depressive symptoms, as measured by the Center for Epidemiological Studies Depression Scale. Browne et al. (2016) found similar results that among barriers of cost and accessibility, drug use stigma stood out as a significant barrier to seeking substance use treatment and improving related negative health outcomes.

If stigma about people who inject drugs is associated with negative health outcomes, then creating a stigma intervention is necessary to counteract the harmful effects. Past work on stigma reduction has focused on informational workshops that disseminate information about the topics of stigma and bias for people who use drugs as an attempt to reduce harmful levels of stigma (Bland et al., 2001; Bahora et al., 2008). Research by Hayes et al. (2004) tested the effectiveness of these informational workshops compared to workshops designed to teach multiculturalism or acceptance and commitment training (ACT). A sample of substance abuse counselors were randomly assigned to one of three workshop conditions and then given measures of stigma immediately post-intervention and at a 3-month follow up. While the multicultural workshop was designed to teach about the benefits of accepting people from other backgrounds by showing their accomplishments in counseling and the value of culturally sensitive treatment, the ACT workshop promoted acceptance and mindfulness about the complex emotions surrounding substance abuse to counteract any negative attitudes toward the stigmatized outgroup. Of all three workshop conditions, the researchers found that the ACT workshop had the greatest reduction of stigma at the 3-month follow up and showed the greatest reduction in negativity for attitudes about stigmatized groups. This suggests that promoting mindfulness and compassion may be more effective in reducing stigma than traditional informational interventions.

Further research by Heath et al. (2018) used self-compassion to moderate the harmful effects of perceived and anticipated public stigma on help seeking. Leary et al. (2007) similarly found that higher levels of self-compassion correlated with fewer negative thoughts and that this self-compassion could be experimentally manipulated. The literature defines self-compassion as a form of empathy for the self that encourages experiencing kindness toward oneself as well as recognizing that individual failures are a shared common human experience and not unique to the individual (Neff, 2003). Heath et al. examined whether a self-compassionate attitude toward oneself would increase one’s resistance to perceived public stigma by creating a buffer of positive affect. This buffer of positive affect could serve as an important technique to address the negative outcomes of stigma for marginalized populations.

The concept of using compassion for oneself is directly related to the concept of empathy. Empathy involves connecting with one’s emotions and relates to the idea of perspective taking or understanding other people’s mental states. If cultivating self-compassion can produce a buffer of positive affect against perceived stigma, then fostering similar types of positive affect through empathy interventions (E.I.) could produce similar results for perceptions of others. Research by Andreychik and Migliaccio (2015) has distinguished between two different types of empathy, both positive and negative. Positive empathy relates to connecting with others’ positive emotions (joy, excitement) while negative empathy relates to connecting with others’ negative emotions (sadness, suffering).

Dovidio et al. (2010) used empathy as a possible way to reduce systematic discrimination and combat stigma against a marginalized out-group. Dovidio et al. cites an Anti-Bias Intervention model to reduce explicit negative associations of an outgroup like people who inject drugs. This model states that by using anti-bias interventions like perspective-taking, one can increase the empathy for a given out-group. Dovidio found that increasing empathy for Blacks, a stigmatized out-group, is associated with lower levels of explicitly measured bias toward Blacks when compared to Whites and improved intergroup attitudes between Blacks and Whites. If increased empathy was connected with lower levels of bias toward an outgroup, Dovidio theorized that an empathy manipulation could affect explicit attitudes on intergroup relations.

The findings of Dovidio et al. are dependent on the ability to identify an individual as a member of an outgroup to measure explicit levels of bias. For an out-group like people who inject drugs, the stigmatizing behavior is difficult to identify compared to more salient markers of stigma like race or physical disability. However, research by Vescio et al. (2003) examined whether the stereotypicality of an out-group influenced the endorsement of negative stereotypes about that outgroup. In their study, half of the participants were given an interview segment where an outgroup member discussed more stereotypical negative experiences suffered as a result of being part of a racial outgroup while the other half of the participants were given an interview segment where the outgroup member discussed general negative experiences. Participants who had seen the stereotypical interview segment rated the entire outgroup as being more negative and stereotypical and reported more negative intergroup attitudes than the other participants. Vescio et al. then added an empathy manipulation in the form of perspective taking to both conditions and told the participants to adopt the perspective of the target interviewee. Participants exposed to the perspective taking intervention reported partially improved intergroup attitudes regardless of which interview segment they had seen. However, the researchers found that situational attributions were a much stronger prediction of improved intergroup attitudes than empathy. If the research suggests fostering empathy in the form of perspective taking could help improve intergroup attitudes, why would empathy not be the most reliable predictor of improved intergroup attitudes?

Cameron et al. (2016) suggest that feelings of empathy for stigmatized groups could be hindered by anticipated emotional exhaustion. They argue that highly stigmatized targets, like people who use drugs, produce high levels of anticipated mental exhaustion due to their membership in a stigmatized group being perceived as highly negative. This anticipated exhaustion leads people to avoid being compassionate and “wasting” their cognitive resources on empathy due to a feeling that it would not be “worth the effort.” This lack of empathetic behavior displayed toward a stigmatized group then allows easier dehumanization and stigmatization as the ingroup avoids feeling guilty over their lack of compassion. Cameron et al. tested this hypothesis by having participants read vignettes of a person who is physically ill (non-stigmatized) and a person who uses drugs (stigmatized), and then rated their levels of perceived emotional exhaustion. As expected, they found that the stigmatized vignettes predicted higher levels of emotional exhaustion. The overwhelming negativity of stigmatizing thoughts about an outgroup like people who use drugs leads to a greater likelihood to actively dehumanize members of this outgroup to save cognitive resources. This limits the effectiveness of empathy manipulations as the empathy manipulations do not change the levels of perceived emotional exhaustion for a target outgroup.

However, research would suggest that the efficacy of empathy manipulations could depend upon the distinction between positive and negative empathy. Andreychik and Migliaccio (2015) showed that there was a distinct difference between negative empathy, identifying with one’s negative emotions, and positive empathy, identifying with one’s positive emotions. Research by Gonzalez et al. (2015) suggest that using positive empathy could provide a more effective empathy manipulation than those previously used to reduce stigma. The researchers argue that positive interactions and feelings toward an outgroup can lower the levels of perceived differences between two groups. They cite the common ingroup identity model (Gaertner et al., 1993), which says that re-categorizing members of an outgroup into one larger group, rather than stigmatized and non-stigmatized subcategories, can prevent the “us” vs. “them” mentality that leads to increased stigmatization. While the research suggests that perspective taking interventions can increase empathetic feelings toward an outgroup (Shih et al., 2013), the logic from Gonzalez et al. suggests that we can use positive empathy to also decrease stigma toward an outgroup. If an intervention is focused on positive emotions, the intervention can create new associations of “outgroup” and “good” rather than the traditional association of “outgroup” and “bad” that is reinforced through negative focused interventions traditionally found in stigma reduction literature.

The literature is clear that stigma against people who inject drugs is associated with negative health outcomes (Keyes et al., 2010; Browne et al., 2016; Paquette et al., 2018). However, reducing that stigma is difficult due to the anticipated emotional exhaustion from helping a heavily stigmatized group (Cameron et al., 2016). The present study seeks to test an intervention to reduce harmful stigma against people who inject drugs by using positive empathy-based perspective taking highlighted in Gonzalez et al. (2015). Creating new associations using positive empathy-based perspective taking could boost the efficacy of empathy-based interventions for stigmatized groups. This new positive focused perspective taking intervention could be more effective at helping to mitigate the harmful effects of widespread stigma against people who inject drugs than previous interventions, as it would allow people to empathize with a stigmatized group without feeling emotionally drained by the experience. We predict that by fostering positive empathy using an imagined positive perspective taking exercise, participants will report significantly lower levels of stigma against people who inject drugs due to new associations of this outgroup and “good.” Specifically, we hypothesize that a positive E.I. will be more effective than a non-informational control condition at reducing stigma and, secondarily, that traditional methods for stigma reduction through learning and information-based interventions will be more effective than a non-informational control condition but less effective than the positive E.I.

## Materials and Methods

### Participants

We recruited 375 subjects through SurveyMonkey.com, a website used to host online surveys and market research. Participants were recruited through SurveyMonkey in order to reach a country-wide sample to develop a generalized intervention. This sample consisted of participants from the United States with a census distribution of gender, age, household income, and religion.

### Study Design

The experiment used a between-subjects design with subjects assigned to one of three conditions: an E.I., a learning intervention (L.I.), and a control. The subjects were recruited in waves with each condition recruited separately due to limitations in the survey software. The independent variable was level of intervention received within each study condition. The dependent variable was the score on a social distance scale (Bogardus, 1925; Link et al., 1987), adapted for the purposes of this study, after being exposed to the assigned study condition.

### Procedure

Participants were contacted through email and invited to complete the study electronically through the SurveyMonkey website. Participants completed the study in three waves with each wave consisting of one condition at a time; the control condition was recruited first, followed by the L.I., and then the E.I. to reduce the likelihood of contamination across waves and address the potential bias resulting from recruitment software limitations. Each wave of survey responses was collected 2 weeks after the previous wave. Participants were told they were taking part in a study meant to measure their attitudes on a wide range of social issues. They were then prompted on the screen to take their time with all the tasks and read the instructions carefully.

Participants in all three conditions were told to read the information given to them on the next screen and that they will be asked questions about what they read. Participants in Arm 1 (E.I.) and Arm 2 (L.I.) were then given the written information from the “State without StigMA” campaign. This information can be found in Supplementary Appendix A. Participants in Arm 3 control intervention (C.I.) were given a written passage from National Geographic. Following the reading of this information, participants in Arm 1 (E.I.) were then given the positive perspective taking exercise and questions designed to engage the participant and prime them with positive empathy. Participants in Arm 2 (L.I.) and Arm 3 (C.I.) were given their respective intervention engagement questions. Participants in Arms 1, 2, and 3 were then given a vignette adapted from Link et al. (1987). This vignette was used previously to act as stigmatized stimuli for participants. Our adapted vignette depicts “Roger Johnson,” and the details of his life like his career status, life goals, and future aspirations. The vignette can be found in Supplementary Appendix B. In addition, the vignette contains one sentence that identifies the subject as a person who injects drugs. This vignette and its subject, “Roger Johnson,” served as the target stimuli to represent the population of people who inject drugs. Participants then completed a social distance scale which includes questions on social distance from people who inject drugs.

After going through their respective conditions, all participants were given a demographic posttest questionnaire, debriefed, compensated $2 for their time, and thanked for their participation. The study procedure was approved by the funding institution’s IRB.

### Materials

#### Arm 1 – Empathy Intervention

Arm 1 was an intervention that used an imagined contact exercise to prime participants with positive empathy. The exercise was adapted from a similar manipulation from Turner et al. (2007). Specifically, the exercise had participants imagine an interaction with an individual who talks about their life and current situation as a person who injects drugs. The participant is then asked a series of open-ended questions designed to encourage positive empathetic engagement. The positive empathy engagement questions were adapted from the ally behavior development methodology of Gonzalez et al. (2015) and similar perspective taking exercises from Saguy et al. (2009) and Joyce and Harwood (2014). Participants were given the following prompt:

You are sitting on a train and a stranger about your age sits down next to you. The stranger is friendly and compliments you on your style. As you strike up a conversation, you learn that the stranger has just started a new job. As you learn more, the stranger discloses to you that they are a person who injects drugs. They tell you about how challenging it was to find employment despite their training and experience in their field. They go on to tell you that they are treated poorly in hospitals and public places and how they are often unfairly discriminated against by other members of society because of their drug use. They tell you that they are often discouraged because people do not see their accomplishments and only see their struggles.

Participants were then given the following prompts designed to prime them with positive empathy:

Can you think of a time when someone failed to see your accomplishments? What differs about your situation compared to the stranger? What would you say to the stranger to make him feel better about his current situation? What kind of positive impact do you think your comments will have on the stranger? How can you make a positive impact on other people like the stranger in the future?

Participants in this condition were also given written information from the “State without StigMA” (What is stigma? The Stigma of Opioid Addiction, 2015) educational campaign from the Massachusetts Department of Public Health. This educational campaign consists of information designed to educate on distinctions between different kinds of stigma, examples of enacted stigma, and suggested actions to prevent stigma for people who use drugs. The information provided in this educational campaign is congruent with a systematic review by Livingston et al. (2012) who found similar campaigns to be effective in reducing stigma.

#### Arm 2 – Learning Intervention

Arm 2 was a L.I. that used traditional methods of stigma reduction to reduce harmful stigma against people who use drugs. In developing a new intervention for stigma reduction for people who use drugs, we sought to test the effects of current programs aimed at reducing these levels of harmful stigma. We took this information from the Massachusetts Department of Public Health’s “State without StigMA” (What is stigma? The Stigma of Opioid Addiction, 2015) campaign to mimic real world applications of these informational learning campaigns used to fight stigma. This is the same written information given to participants in Arm 1. Following this, participants were also given a prompt to write down five things they remembered from the previous screen to control for engagement with the intervention materials.

#### Arm 3 – Control Intervention

Arm 3 was a C.I. which served as a baseline to measure the effectiveness of the E.I. in Arm 1 and the L.I. in Arm 2. Participants in Arm 3 were given information about the rainforest pulled from the transcript of a National Geographic documentary and given relevant engagement prompts. Similar to Arms 1 and 2, these answers were taken in open response format to control for attention and engagement of the previous written material.

### Measures

The social distance scale was used to measure feelings of comfort for several different scenarios regarding our target outgroup. In measuring outcomes for sensitive topics like stigma that might carry a social desirability bias to answer in a particular way, previous studies have used social distance as an indirect way to measure intentions for stigmatizing behavior to collect more accurate attitudinal data (Ashford et al., 2018; Drake et al., 2018). The scale used in the current study is adapted from previous studies measuring social distance (Bogardus, 1925; Link et al., 1987) and has been extensively replicated and validated as a reliable measure (Newcomb, 1950; Hartley and Hartley, 1952; Sherif and Sherif, 1956). All social distance questions were about “Roger Johnson,” our target stimuli representing the population of people who inject drugs. Past studies have studied social distance for target populations on different metrics like race, economic status, and mental health. The current study measured social distance for people who inject drugs. This use case is supported by a pilot study from Ashford et al. (2018) measuring social distance for individuals with a substance use disorder.

The adapted social distance scale in the current study measures the comfort level of subjects on a scale of 0 (definitely willing) to 5 (definitely unwilling) for sixteen scenarios with select scenarios being reversed scored. For example, participants would rate their comfort level with renting a room to a person who injects drugs, being a neighbor to a person who injects drugs, or allowing their children to play outside if a person who injects drugs lived nearby. Results on the scale were averaged to create one social distance score. A higher average social distance score is correlated with a higher perceived desire for greater social distance from the target outgroup. The full social distance scale can be found in Supplementary Appendix C.

Participants also completed a demographic posttest after the completion of the other materials. This posttest included questions about age, gender, household income, and religion.

### Data Analysis

Arm 1 (E.I.) and Arm 2 (L.I.) required reading information to adequately engage with the intervention materials. The average response time in Arm 1 and Arm 2 was approximately 11 min and the average response time in Arm 3 was approximately 7 min. Response times that were less than 3 min indicated insufficient engagement with the intervention materials and those participants were disqualified from the study for not following the protocol.

All data analysis was completed via IBM SPSS v25. Statistical significance for all tests was defined at *p* = 0.05. Analysis of the stigma scores was completed for each participant group using one-way analysis of variance (ANOVA) tests with social distance as the dependent variable and condition as the independent variable. A separate ANOVA test was used to compare posttest demographic information of participants in all three conditions for significant differences with the independent variables being age, gender, household income, and religion and the dependent variable being average stigma score.

We conducted an *a priori* power analysis of the effect size corresponding to the difference in empathy scores between experimental groups using G power software (Faul et al., 2007). The desired sample size for each group was *n* = 96 for a medium effect size target at *p* < 0.05 which is consistent with the significance from previous work using similar methods and group sizes (Ashford et al., 2018; Witte et al., 2018).

## Results

A total of 375 participants were recruited for this study with Arms 1, 2, and 3 all having equal participants (*n* = 125). Low response times resulted in excluding subjects from Arm 1 (*n* = 11), Arm 2 (*n* = 14), and Arm 3 (*n* = 8), leaving *n* = 342 for analysis. Overall our sample was 58% female with a majority of participants identifying as White (74%), followed by African American (16%), Asian/Pacific Islander (8%), and other (2%). There were no statistically significant differences found between subjects across all three arms at baseline.

The average stigma score data was consistent with our primary hypothesis that average stigma scores measured by the post-test social distance questionnaire would be lowest in Arm 1 (E.I.). Arm 1 had the lowest average stigma score (3.49) and this score was significantly lower than the average stigma score of Arm 3 (C.I.) (3.69), with a mean score of *F*(114) = 3.49, *p* = 0.01. With regard to our secondary hypothesis, we did not find a statistically significant difference between the average stigma score of Arm 2 (3.56) and Arm 3 (3.69) although the data trended toward significance, *F*(111) = 3.56, *p* = 0.167. Notably, there was no statistically significant difference between the Arm 2 stigma reduction materials (3.56) and control Arm 1 [*F*(114) = 3.49, *p* = 0.51].

On a measurement of post-test demographics, we found no significant relationships between stigma score and age, gender, household income, or religion. A breakdown of demographic variables on average stigma score can be seen in Supplementary Appendix D.

## Discussion

Supporting our primary hypothesis, we found that a positive imagined contact E.I. significantly reduced post-test levels of held social distance when compared to participants that were not exposed to any stigma reduction intervention. These findings are consistent with the logic from past literature on perceived emotional exhaustion (Cameron et al., 2016) and the effectiveness of positive empathy-based perspective taking (Gonzalez et al., 2015) and contribute to a growing body of research looking at positive empathy as a distinct and effective construct to mitigate harmful effects of stigma. Contrary to our secondary hypotheses, we did not find a significant difference between our E.I. and the publicly available stigma L.I., although there was a trend toward statistical significance, and there was no significant difference between the L.I. and the control.

Past literature has shown that despite attempts to curtail the negative effects of stigma, people who inject drugs are still suffering negative health outcomes when seeking treatment (Latkin et al., 2010; Paquette et al., 2018). Our hypothesis was that one of the problems in addressing that stigma to prevent negative health outcomes was the anticipated negativity from dealing with a marginalized population (Cameron et al., 2016). To address this concern, our vignettes about the stigmatized group use positive associations of the outgroup and good. By using positive focused stimuli to prevent the activation of anticipated negativity, we create the opportunity to form new positive associations which could help explain our significant reduction in stigma scores between the positive E.I. and the control condition.

Similarly, the lack of significant reduction in stigma between the traditional L.I. and the control can be explained using similar logic. While the positive E.I. presents positive empathy in the form of perspective taking, the L.I. uses only information-based presentation of stigma literature. Without this positive focused empathy component, it is possible that there is no significant reduction in stigma for people who use drugs due to anticipated negativity of reading or interacting with the target stigmatized population presented in the information-based intervention. While there may be some impact from the L.I. compared with the control condition, this could be explained by a recency effect of having read information about stigma literature from the intervention itself with no deeper processing of the mechanisms of stigma or bias needed for a longer lasting stigma reduction effect. Our study suggests that the traditional seminar-based stigma interventions often employed in communities and workplaces are a good start but require deeper emotional processing of stigma through interventions that seek to counteract the harmful associations prevalent in those places.

One important consideration when developing future empathy-based stigma interventions is the use of imagined intergroup contact in place of real interpersonal contact between groups. Previous literature has shown the importance of intergroup contact to promote positive intergroup behavior (Saguy et al., 2009; Joyce and Harwood, 2014). However, not every environment is conducive to in-person intergroup contact. The findings of Turner et al. (2007) and Joyce and Harwood (2014) suggest that the benefits of intergroup contact are also found when that contact is hypothetical or imagined. While a limitation to finding stronger effects from more emotionally charged interactions between groups, imagined positive perspective taking exercises are important to address intergroup stigma in scenarios where in person contact is a logistical challenge.

### Limitations

While the sampling from SurveyMonkey provided us with a diverse and generalizable sample for our target demographic with no significant differences, there were technological limitations to the recruitment resulting in the lack of random assignment for the three study conditions and an overall non-rigid study design. In addition, the current study did not measure pre-intervention levels of social distance for the three study conditions due to concerns of contamination that could arise from taking a bias measure before the experimental material in each condition. Future studies should consider testing randomly assigned participants across conditions and for pre-intervention levels of social distance to control for any differing levels of social distance at baseline. While our results were significant (*n* = 342, *p* = 0.01) for the E.I., a larger sample size using more participants in a robust research participant pool would be able to draw conclusion more fully about the differential efficacy of these interventions. The current study did not follow-up to assess the durability of our stigma reduction effects for our target population, which is also an important area for future study.

### Future Directions

One important benefit to focusing on positive empathy-based interventions is the creation of scenarios and examples in which the outgroup being studied is not being painted in a positive and victimless light. If one of the problems with studying stigma and empathy for marginalized groups is the constant perceived emotional exhaustion from their communities for interaction with these groups, then creating new associations and scenarios showing these marginalized groups in a positive light could help to combat these overwhelmingly negative associations of these marginalized groups through more positive focused emotional stimuli. An additional benefit of positive-focused intervention materials is the shift from deficit-based stigma research to desire based stigma research to better facilitate and foster feelings of positive empathy. Further research could examine whether our positive empathy technique shows significant stigma reduction as a standalone measure or if the shift in focus away from deficit-based stigmatization materials through positive empathy can be utilized in traditional stigma reduction techniques to help display significant effects. Future study into stigma and empathy should seek to test positive E.I. with larger sample sizes as well to create a foundation of positive empathy focused intervention work that is lacking in the literature.

Our study sought to test our novel intervention on a sample of the general population as it is important to reduce stigma at the community level where that perception is constantly felt by marginalized populations (Fisk and Neuberg, 1990; Judd and Park, 1993; Link and Phelan, 2001). One possible avenue of future research could look at testing the intervention in specific populations that deal more directly with people who use drugs. Research by Paquette et al. (2018) examined the negative health outcomes from stigma at the macro-level in healthcare services for people who inject drugs. Testing our positive E.I. for healthcare workers who deal directly with this marginalized population could yield interesting results on the efficacy of stigma reduction techniques in a practice setting to reduce stigma from the population that has the most repeated contact with this marginalized group. If effective, our novel intervention could be deployed in several different healthcare and community settings to reduce the harmful effects of stigma on people who use drugs. Overall, our study suggests that the use of imagined positive perspective taking exercises is a promising strategy to create new positive associations of people who use drugs that offers more promising and flexible applications than traditional information-based stigma interventions. Further research on positive empathy-based stigma reduction techniques is needed to test best methods and applications of empathy to reduce stigma directed at marginalized populations.

## Data Availability Statement

The raw data supporting the conclusions of this article will be made available by the authors, without undue reservation.

## Ethics Statement

The studies involving human participants were reviewed and approved by West Virginia University IRB. The patients/participants provided their written informed consent to participate in this study.

## Author Contributions

AC conducted the study, collected and analyzed the data, and wrote the manuscript. RP advised on study procedure and edited the manuscript. Both authors contributed to the article and approved the submitted version.

## Conflict of Interest

The authors declare that the research was conducted in the absence of any commercial or financial relationships that could be construed as a potential conflict of interest.
