# Supplementary material for: Using Positive Empathy Interventions to Reduce Stigma Toward People Who Inject Drugs
Source: Front Psychol. 2021 Jul 9;12:616729. doi: 10.3389/fpsyg.2021.616729 (PMC8298892; doi:10.3389/fpsyg.2021.616729)
Supplement: Supplementary file 1 [file Table_1.docx]

**Appendix A**

**‘State without StigMA’: The Stigma of Opioid Addiction**

People who struggle with addictions to prescription painkillers and heroin (known as opioids) face a wide range of stigmas. A stigma is a mark of disgrace that sets a person or a group apart. 

When people are labeled primarily because of their addiction, they are being negatively stereotyped. Biased, hurtful words, attitudes and behavior represent prejudices against people with substance use disorder, and often lead to their discrimination and social exclusion.

Stigmas can also create physical and mental barriers for people with addiction to seeking treatment.

**Personal, Social and Institutional Stigma**

Stigmas aimed at people with substance use disorders come from many sources. In recent research*, people in recovery from addiction have identified the following examples:

- **Personal.** Self-disgust, shame and self-hate at one’s own appearance, behavior, lifestyle and/or physical condition, as well as feelings of being unworthy of help or recovery
- **Social.** Negative perceptions, labels and actions from friends or family; feeling isolated or rejected
- **Institutional.** Negative treatment and attitudes experienced from healthcare providers, the media, law enforcement, places of work or government agencies

**Examples of Stigma**

Recent interviews with people in recovery from opioid addiction report the following examples of stigma*:

- Hurtful words and labels, including junkie, loser, thief, druggie, abuser and addict
- Comments, such as “Once a junkie, always a junkie” or “You’re not fit to be a parent”
- Perceptions, including:
  - Addiction is a personal choice (when in fact it’s a disease)
  - Addiction is a sign of human weakness, or a lack of morals or willpower
  - Addiction is the result of poor parenting.
- People in recovery with children have experienced other parents unwilling to let their children play at their schoolmate’s homes
- Some communities view addiction as a crime, an act that must be penalized, versus a disease that needs treatment
- People known to local law enforcement have reported being “profiled”

**Addiction is a Disease**

Many science and medical practitioners have concluded that addiction is a chronic, often relapsing disease of the brain. Addiction in some cases may have genetic roots. Although the initial decision to take drugs may be voluntary, chemical and neurological changes to the brain severely restrict a person’s self-control. The disease hinders one’s ability to resist intense impulses to take drugs – despite harmful consequences to the addicted individual and to those around him or her.**

**What Can We Do?**

It’s important to understand that opioid addiction is a treatable disease. On a personal level, we can look honestly at ourselves for signs of our own stigmas – negative, biased feelings, words or behaviors. We can make the effort to look at addiction from a different and hopefully more positive perspective. 

We are all affected by the current epidemic of opioid addiction. Many people know someone who struggles with addiction, or who is in treatment or recovery.

- We can all be part of the solution
- We can take a stand against stigma
- We can support treatment opportunities
- We can encourage people in recovery
- Most importantly, we can talk about addiction amongst our friends and family members to hopefully address the misperceptions about addiction, treatment options and long-term recovery
- Each of us can commit to not using hurtful or damaging words about those who face addiction

* 2015 research on stigma for people with addiction disorders conducted by the Massachusetts Bureau of Substance Abuse Services, Department of Public Health, Executive Office of Health and Human Services.

**Appendix B**

**Current Use Vignette Adapted from Link et al. 1987**

Here is a description of a 27-year-old man, let's call him Roger Johnson. Roger works hard during the week and injects heavy drugs on the weekend. Roger works at a job in a local business. He earns $35,000 a year before taxes and is doing well enough. He is well groomed and known for dressing neatly. At his job, he gets along well with his co-workers and is on friendly terms with them. He begins his days chatting briefly with the people he works with and then gets down to business. He takes coffee and lunch breaks during the day, just like everyone else, and returns to work when his co- workers do. While on the job, Roger checks his work carefully and doesn't pass it along until it is correct. This might slow Roger down a little, but he is never criticized for the quality of the work he completes. Roger is interested in meeting and dating young women in the community. He is considering joining a local church group to meet them. He is also looking for a job that gives him more responsibility and pays better than his current one.

**Appendix C**

**Adapted Social Distance Items**

1. I would feel comfortable renting a room in my home to someone like Roger Johnson.

2. I would not feel comfortable being a worker on the same job as someone like Roger Johnson. (Reversed Scoring).

3. I would feel comfortable having someone like Roger Johnson as a neighbor.

4. I would feel comfortable leaving Roger Johnson as the caretaker of my children for a couple of hours.

5. I would feel comfortable having one of my children marry someone like Roger Johnson.

6. I would feel comfortable introducing Roger Johnson to a young woman I am friendly with.

7. I would not feel comfortable recommending someone like Roger Johnson for a job working for a friend of mine. (Reversed Scoring)

8. I would not feel comfortable hiring someone like Roger Johnson to work for myself. (Reversed Scoring)

9. If a group of people who formerly injects drugs lived nearby, I would not allow my children to go to the movie theater alone. (Reversed Scoring)

10. If a person who formerly injects drugs applied for a teaching position at a grade school and was qualified for the job I would recommend hiring him or her.

11. One important thing about people who inject drugs is that you cannot tell what they will do from one minute to the next. (Reversed Scoring)

12. If I know a person had problems with injection drug use, I will be less likely to trust him. (Reversed Scoring)

13. The main purpose of drug treatment programs should be to protect the public from people who use drugs. (Reversed Scoring)

14. If a person who formerly injects drugs lived nearby I would not hesitate to allow young children under my care to play on the sidewalk.

15. Although some people who inject drugs may seem all right it is dangerous to forget for a moment that they are users. (Reversed Scoring)

16. There should be a law forbidding a person who formerly injects drugs the right to obtain a driver’s license. (Reversed Scoring)

Response format 0 = strongly agree, 1 = agree, 2 = not sure but probably agree, 3 = not sure but probably disagree, 4 = disagree, 5 = strongly disagree.

**Appendix D**

**Average stigma score broken down by demographic variable, total n=342.**

| **Demographic Variable** | **Number** | **Avg. Stigma Score** | **Std. Deviation** |
| --- | --- | --- | --- |
| Age |  |  |  |
| 18-29 | 98 | 3.5189 | .57597 |
| 30-44 | 78 | 3.5962 | .58015 |
| 45-60 | 120 | 3.6429 | .47081 |
| > 60 | 46 | 3.5590 | .43024 |
| Gender |  |  |  |
| Male | 143 | 3.6129 | .49519 |
| Female | 199 | 3.5656 | .54434 |
| Household Income |  |  |  |
| $0-$9,999 | 16 | 3.5402 | .44337 |
| $10,000-$24,999 | 46 | 3.5388 | .53297 |
| $25,000-$49,999 | 78 | 3.5100 | .54977 |
| $50,000-$74,999 | 63 | 3.6916 | .46660 |
| $75,000-$99,999 | 50 | 3.5200 | .66492 |
| $100,000-$124,999 | 29 | 3.7044 | .38641 |
| $125,000-$149,999 | 17 | 3.7059 | .44095 |
| $150,000-$174,999 | 6 | 3.4405 | .69608 |
| $175,000-$199,999 | 5 | 3.8429 | .30051 |
| $200,000+ | 8 | 3.7857 | .40225 |
| Prefer not to answer | 24 | 3.4940 | .47376 |
| Region |  |  |  |
| New England | 17 | 3.6050 | .47456 |
| Middle Atlantic | 32 | 3.3951 | .69371 |
| East North Central | 53 | 3.5511 | .53772 |
| West North Central | 20 | 3.7143 | .54201 |
| South Atlantic | 69 | 3.6170 | .46710 |
| East South Central | 20 | 3.5214 | .41137 |
| West South Central | 40 | 3.6554 | .50245 |
| Mountain | 26 | 3.6209 | .43226 |
| Pacific | 60 | 3.5976 | .56165 |
